# Telepsychiatry and Artificial Intelligence: A Structured Review of Emerging Approaches to Accessible Psychiatric Care

**DOI:** 10.3390/healthcare13111348

**Published:** 2025-06-05

**Authors:** Artem Bobkov, Feier Cheng, Jinpeng Xu, Tatiana Bobkova, Fangmin Deng, Jingran He, Xinyan Jiang, Dinislam Khuzin, Zheng Kang

**Affiliations:** 1 School of Health Management, Harbin Medical University, Harbin 150081, China; bobkov.a.m@yandex.com (A.B.); chengfeier0830@163.com (F.C.); jinpeng9789@gmail.com (J.X.); derfmin@163.com (F.D.); jingran619@163.com (J.H.); 15245390831@163.com (X.J.); 2 Department of Rheumatology and Immunology, The Second Affiliated Hospital of Harbin Medical University, Harbin 150001, China; tat.voropaeva.1993@mail.ru; 3 Department of General Chemistry, Bashkir State Medical University, Ufa 450000, Russia; dinislamkhuzin@mail.ru

**Keywords:** artificial intelligence, telepsychiatry, digital psychiatry, machine learning, AI ethics, remote diagnostics, digital interventions, translational research

## Abstract

Background/Objectives: Artificial intelligence is rapidly permeating the field of psychiatry. It offers novel avenues for the diagnosis, treatment, and prediction of mental health disorders. This structured review aims to consolidate current approaches to the application of AI in telepsychiatry. In addition, it evaluates their technological maturity, clinical utility, and ethical–legal robustness. Methods: A systematic search was conducted across the PubMed, Scopus, and Google Scholar databases for the period spanning 2015 to 2025. The selection and analysis processes adhered to the PRISMA 2020 guidelines. The final synthesis included 44 publications, among which 14 were empirical studies encompassing a broad spectrum of algorithmic approaches—ranging from neural networks and natural language processing (NLP) to multimodal architectures. Results: The review revealed a wide array of AI applications in telepsychiatry, encompassing automated diagnostics, therapeutic support, predictive modeling, and risk stratification. The most actively employed techniques include natural language and speech processing, multimodal analysis, and advanced forecasting models. However, significant barriers to implementation persist—ethical (threats to autonomy and risks of algorithmic bias), technological (limited generalizability and a lack of explainability), and legal (ambiguous accountability and weak regulatory frameworks). Conclusions: This review underscores a growing disconnect between the rapid evolution of AI technologies and the institutional maturity of tools suitable for scalable clinical integration. Despite notable technological advances, the clinical adoption of AI in telepsychiatry remains limited. The analysis identifies persistent methodological gaps and systemic barriers that demand coordinated efforts across research, technical, and regulatory communities. It also outlines key directions for future empirical studies and interdisciplinary development of implementation standards.

## 1. Introduction

Mental disorders represent one of the most urgent and destructive challenges confronting global public health in the 21st century. According to the World Health Organization (WHO), anxiety disorders alone had already affected more than 300 million people by 2019. The situation deteriorated dramatically during the COVID-19 pandemic. Within a single year, the prevalence of anxiety and depressive symptoms surged by 25%. As of early 2023, depression had been diagnosed in 280 million individuals—approximately 4% of the global population [1,2]. These conditions not only compromise quality of life and social functioning but also impose a profound economic burden: an estimated 12 billion workdays are lost annually, costing the global economy over one trillion USD [3].

The crisis is further compounded by a pronounced shortage of qualified mental health professionals, particularly in low-resource settings and remote regions where psychiatric care is often entirely unavailable. According to a WHO report, the median global psychiatrist density in 2020 was 13 per 100,000 population, with high-income countries exceeding the rate observed in low-income regions by more than fortyfold [4]. Even in the United States—home to one of the world’s most developed healthcare systems—a shortfall of over 17,000 psychiatrists is projected by 2030 [5]. In the United Kingdom, nearly one-third of child psychiatry positions are currently unfilled [6]. Several European countries fare no better, with fewer than one psychiatrist available per 10,000 inhabitants [7]. These data collectively underscore the urgent need to reconsider existing models of psychiatric service delivery.

In response to these challenges, the digital transformation of healthcare—particularly the integration of artificial intelligence (AI)—is increasingly viewed as a strategic axis of development [8,9]. In psychiatry, AI has already been employed for speech and affective behavior analysis, suicide risk prediction, and psychometric monitoring via mobile applications, as well as for remote support through chatbot-assisted interactions [10,11]. Telepsychiatric platforms based on machine learning and the concept of “digital phenotyping” are especially promising, enabling the design of personalized therapeutic strategies and expanding access to care in resource-limited regions [12]. However, this potential is far from self-actualizing. The use of AI in psychiatry faces a constellation of challenges that extend well beyond the technological domain. A critical gap remains between the laboratory precision of AI models and their real-world clinical applicability—a gap that must be acknowledged and strategically bridged.

Over the past decade, only a limited number of reviews have addressed the intersection of artificial intelligence and telepsychiatry, with most being either outdated or narrowly focused. For example, the scoping review by T. & Annamalai (2020) [13] summarized early conceptual developments but included literature only up to mid-2020 and did not assess methodological quality. Subsequent narrative reviews have concentrated on isolated aspects—AI-based chatbots [14], traditional telepsychiatric processes [15], or generative language models [16]—while overlooking multimodal architectures and regulatory shifts. To the best of our knowledge, no structured review published since 2021 has systematically examined AI applications across all stages of telepsychiatric care—diagnosis, monitoring, therapeutic support, and prediction—while simultaneously assessing technological maturity and ethical–legal robustness. This review seeks to fill that critical gap by offering a comprehensive, PRISMA-aligned synthesis of the literature, with a focus on translational barriers, system readiness, and governance of digital interventions. While there has been substantial interest in the technological progress of artificial intelligence, the majority of reviews limit themselves to describing possibilities without adequately addressing the primary barriers to scaling and integration into clinical practice. This study is dedicated to a systematic analysis of these translational challenges and outlines practical steps necessary for transitioning from experimental models to clinically robust tools in telepsychiatry. 

**Figure 1:**
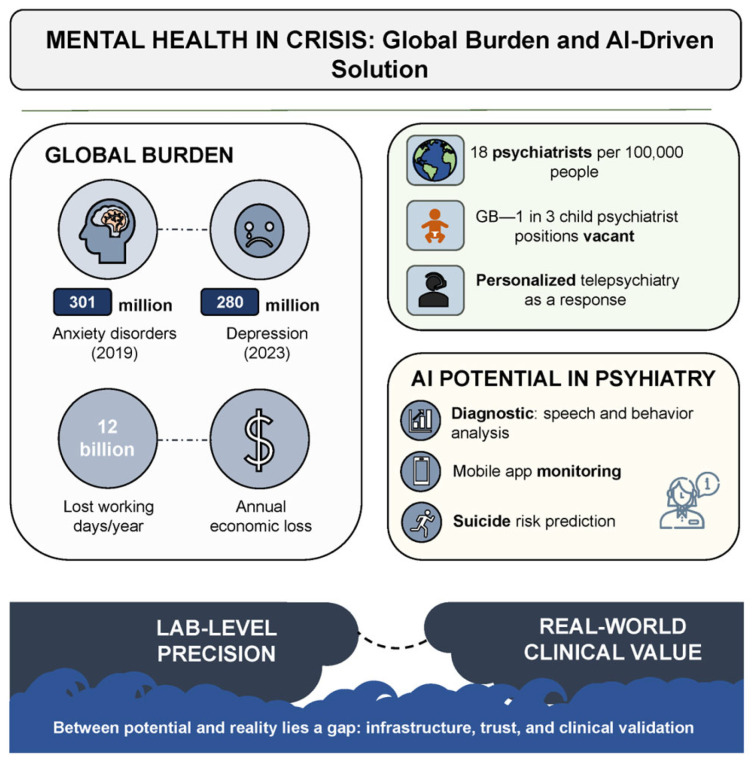
Mental health in crisis: global burden and AI-driven solution.

Figure 1 schematically illustrates how the convergence of critical challenges—including workforce shortages, rising demand, and massive economic losses—creates a “crisis zone” in which innovative solutions are no longer optional but imperative.

Despite the growing interest in the application of artificial intelligence in psychiatry, its use within telepsychiatry remains fragmented and insufficiently systematized. Existing reviews tend to focus either on digital platforms or machine learning algorithms, often neglecting the distinct challenges of remote mental health care and the nuances of clinical context.

Bridging foundational innovation and clinical relevance, this review delivers a structured analysis of artificial intelligence in telepsychiatry. It encompasses the full technological range—from classical machine learning methods (support vector machines, logistic regression, random forests, and boosting) to advanced deep learning architectures, such as transformers, BERT, GRU/LSTM, and hybrid models. Special attention is given to natural language processing for speech and text analysis, state-of-the-art tools for explainability and algorithmic fairness (integrated gradients, SHAP, and reweighing), as well as digital assistants, chatbots, federated learning, and privacy-preserving techniques. Ethical and regulatory aspects are addressed through the frameworks of GDPR, LINDDUN, and living-lab models.

This comprehensive yet focused overview provides a nuanced perspective on the current AI ecosystem in psychiatry, forming a basis for assessing technological maturity and clinical applicability. By classifying algorithms and clinical cases, the review highlights key barriers to implementation and research gaps hindering clinical translation, with a distinct emphasis on the interplay of technical, ethical, and regulatory factors shaping the future of digital psychiatry.

The aims of this review are threefold: (1) to synthesize existing scientific evidence on the use of AI in psychiatric practice; (2) to delineate the primary ethical, technological, and legal obstacles; and (3) to outline pathways toward more accessible and effective global mental health care.

## 2. Methodology

### 2.1. Aim and Rationale for the Chosen Format

In line with the stated objectives, the review was initially conceived as a narrative synthesis. However, as empirical studies with reportable quantitative metrics were progressively incorporated, the structure evolved toward that of a systematic review. While a full formalization of all components of a systematic review—such as protocol registration (e.g., in PROSPERO)—was not originally intended, the core stages, including eligibility criteria formulation, search strategy, screening procedures, and data analysis, were subsequently formalized in accordance with PRISMA 2020 recommendations [17].

### 2.2. Sources and Databases

The systematic search was conducted across the international bibliographic databases PubMed and Scopus, with Google Scholar used as a supplementary source to identify non-standard publications and preprints. The search covered the period from 1 January 2015 to 25 March 2025, with a particular emphasis on studies published between 2023 and 2025. These sources were selected due to their relevance and representativeness for the field of digital psychiatry and artificial intelligence. Databases such as Embase and Cochrane were excluded due to substantial overlap with the already covered sources. In addition, a manual search was performed using reference lists from the included studies, recent thematic reviews, and official sources (e.g., WHO, UNESCO, AMA, and Royal College of Psychiatrists, among others).

Grey literature—including dissertations, reports, and conference proceedings—was deliberately excluded, with the exception of preprints and official publications from international organizations that were openly accessible and provided clear methodological descriptions.

### 2.3. Search Strategy

A tailored search strategy was developed for each database, incorporating Boolean operators (AND, OR), domain-specific terminology, and field-specific filters (Title, Abstract, Keywords, and MeSH). The search was structured around three core conceptual domains: (1) artificial intelligence, (2) mental health, and (3) digital psychiatry and telemedicine. The final search iteration was completed on 25 March 2025.

As an example, the search query applied to PubMed is presented below (targeting the Title/Abstract/MeSH fields). In addition to the listed terms, filters were applied for publication date (1 January–25 March 2025), language (English), and sample type (Humans), which may introduce a potential language bias (see Box 1).

Box 1 **Example of PubMed search query.**

(“Artificial Intelligence”[Mesh] OR “Machine Learning”[Mesh]

OR “Deep Learning” OR “Natural Language Processing” OR AI OR ML)

AND

(“Psychiatry”[Mesh] OR “Mental Health” OR Depression OR Anxiety)

AND

(“Telemedicine”[Mesh] OR Telepsychiatry)

AND

(“1 January 2018”[Date—Publication]: “25 March 2025”[Date—Publication])

A separate search strategy was applied for Google Scholar, based on manually curated keyword phrases without the use of formal filters. Search queries were constructed by hand, with an emphasis on identifying non-standard publications, preprints, and thematically relevant sources. Result rankings were determined automatically by the platform, while selection was performed manually through the screening of titles and abstracts. Examples of key phrases used are provided in Supplementary Table S1 (Google Scholar section). All the retrieved records were imported into Zotero (v6.0.30), where duplicate entries were automatically removed. A two-stage screening process followed, consisting of an initial relevance check based on titles and abstracts, and a subsequent full-text evaluation. At each stage, selection was carried out independently by two reviewers; disagreements were resolved through discussion until consensus was achieved.

### 2.4. Inclusion and Exclusion Criteria

Original studies were eligible for inclusion if they applied artificial intelligence methods within psychiatry or closely related disciplines. To meet the criteria, studies were required to report methodological details, describe sample characteristics, and present at least one quantitative performance metric.

Systematic and scoping reviews were also considered, provided they employed a formalized analytical framework.

Only English-language publications released between 2015 and March 2025 were eligible and only if their content aligned with the thematic scope of this review.

Exclusion criteria encompassed studies lacking an AI component, methodological transparency, or quantitative indicators. Additional exclusions included non-full-text materials, opinion pieces, letters to the editor, and publications in languages other than English.

Final inclusion decisions were made independently by two reviewers, in accordance with predefined criteria (see Supplementary Table S2).

### 2.5. Study Selection Procedure

The initial search yielded 4812 unique records. Additional sources of grey literature were also considered: websites (*n* = 14), organizational documents (*n* = 6), and backward citation chains (*n* = 5). However, all of these were directly merged into the main corpus and not processed separately. Deduplication using Zotero (v6.0.30) reduced the dataset to 3225 entries. The initial screening, based on titles and abstracts, was conducted independently by two reviewers and resulted in the exclusion of 3093 records that failed to meet the predefined relevance criteria, including topic, document type, language, and publication date.

At the full-text assessment stage, 132 publications were reviewed. Of these, 94 were excluded due to the absence of quantitative metrics, the lack of methodological detail, or a primary focus unrelated to the application of AI in psychiatry. The final selection comprised 44 publications, including 14 empirical studies reporting explicit performance metrics for AI models. A step-wise exclusion log is summarized in Supplementary Table S2. The remaining 24 studies were included for contextual and normative–ethical analysis.

Screening at all stages was conducted in parallel by two independent reviewers; discrepancies were resolved through discussion until consensus was reached. Formal inter-rater reliability measures (e.g., Cohen’s kappa) were not applied due to the limited volume and high initial agreement between reviewers. All decisions were made following a structured selection procedure. The literature selection process is depicted in the PRISMA 2020 flow diagram (see Supplementary Figure S1).

In addition to the core PRISMA criteria, the study selection also considered the diversity of methodological approaches and geographic representation. Particular attention was given to the variety of AI model types, target mental health conditions, and levels of technological maturity. Priority was assigned to studies providing detailed accounts of algorithmic architectures, performance metrics, and model limitations, which subsequently enabled thematic classification and critical appraisal of the literature.

### 2.6. Compliance with Transparency Principles

Data extraction was performed manually using standardized Microsoft Excel (v. 2108, Build 14332.20721) spreadsheets. This process was applied exclusively to the 15 empirical studies that met the criteria for comprehensive reporting. The extracted parameters included model architecture and type, sample characteristics, and quantitative performance metrics. Review articles were used primarily for contextual and thematic analysis; no formalized data extraction was applied to these sources. Their inclusion was justified by methodological rigor and thematic relevance.

To ensure methodological rigor and transparency, a structured assessment of potential sources of bias was performed for each included study. The evaluation encompassed sample size and representativeness, the presence of blinding or control groups, external validation procedures, outcome assessment methods (both objective and self-reported), data completeness, and disclosure of conflicts of interest. Limitations and risk-of-bias considerations are summarized in Supplementary Table S3 and discussed in detail in Section 4.2.

During the preparation of the manuscript, ChatGPT (OpenAI, GPT-4o mini, v.1.2025.139.0) was used exclusively for stylistic refinement and phrasing alignment. All scientific decisions and conclusions were formulated by the authors; no AI tools were employed for data analysis or result interpretation. All the content related to scientific reasoning and argumentation was developed and edited manually.

## 3. Results

### 3.1. Empirical Studies and Model Characteristics

From each included empirical study, key parameters were extracted: model architecture, data type, performance metrics, and methodological features. A standardized summary of these elements is provided in Supplementary Table S3.

Model architectures ranged from classical approaches (e.g., SVM and logistic regression) to advanced implementations based on GRU, Transformer, and federated learning. The input data spanned audio, text, visual and behavioral signals, sensor outputs, and electronic health records. Several studies conducted external validation procedures, confirming the robustness of the models.

Despite the heterogeneity of study designs, most models demonstrated high performance metrics—such as accuracy, sensitivity, and area under the curve (AUC)—comparable to clinical benchmarks. Limitations of individual approaches (e.g., small sample sizes and a lack of validation) are addressed in Section 4.2. All the studies were categorized according to their application domains and are presented in the corresponding subsections.

### 3.2. Applications of AI in Psychiatric Practice

#### 3.2.1. Diagnosis and Screening

Among the most widespread and effective approaches currently employed are natural language processing (NLP) techniques, which enable the automatic extraction of semantic and emotional features from patients’ spontaneous speech, written narratives, and medical documentation. Contemporary diagnostic algorithms built on technologies such as transformers, bidirectional long short-term memory networks (BiLSTM), gated recurrent units (GRUs), and parallel convolutional neural networks (CNNs) have demonstrated high levels of effectiveness.

For instance, in a study involving 270 participants, a support vector machine (SVM)-based model trained on deep speech features achieved a diagnostic accuracy of 94.1%, with sensitivity and specificity reaching 96.7% and 90.7%, respectively [18]. The highest performance was observed when analyzing spontaneous speech collected during psychiatric interviews, underscoring the importance of naturalistic context in patient assessment. However, the model was evaluated exclusively under internal cross-validation conditions, with no external testing, which limits its generalizability beyond the laboratory setting.

The review represents one of the few systematic attempts to synthesize multimodal (CNN, RNN, BiLSTM, and SVM) approaches that integrate auditory, visual, and textual signals for automated depression detection. Drawing on datasets such as DAIC-WOZ, AVEC, MMDA, and D-Vlog, the authors report accuracy values ranging from 65% to 94.8%, with the highest performance observed in models combining facial and vocal inputs. The survey underscores two consistent findings: multimodal fusion architectures consistently outperform single-channel baselines, and generalizability remains constrained by small, heterogeneous datasets and the lack of standardized evaluation frameworks [19].

Another promising model—TCC (Transformer combined with Parallel CNNs)—demonstrated high performance, achieving both elevated accuracy and resilience to computational resource constraints across multiple datasets. The reported F1-scores reached 93.6% on DAIC-WOZ and 96.7% on MODMA, significantly outperforming baseline models [20]. However, both datasets were used exclusively for internal evaluation without external validation, and participant-level data partitioning may not have been enforced, increasing the risk of overfitting. Despite its architectural novelty and computational efficiency, the model remains experimental and is not yet suitable for clinical deployment.

Contemporary diagnostic approaches increasingly incorporate visual cues—particularly fine-grained facial dynamics and gaze patterns. Mahayossanunt et al. (2023) introduced a Window-Block LSTM with attention and label smoothing that analyzes head pose, eye orientation, and facial action units to detect depressive states. Tested on an internal split of 474 videos (134 moderate–severe depression; 340 non-depressed), the model achieved accuracy = 91.7% and F1 = 88.9%; precision and recall reached 91.4% and 87%, respectively. Although the classifier offered post hoc explainability via integrated gradients—highlighting clinically plausible cues such as downward gaze and reduced brow movement—its generalizability remains uncertain due to the absence of external validation and the small proportion of severe cases [21]. However, validation was limited to a single center with a methodologically homogeneous sample, and causal inferences regarding treatment outcomes were not supported by randomized controlled trials. While the technology shows strong potential, it remains confined to the research setting. Generalized characteristics of the models, maturity levels, and limitations are presented in Table 1.

Thus, despite the high accuracy demonstrated by various models, large-scale implementation remains constrained by a number of fundamental limitations. According to a review of 115 studies, most NLP-based models in neuroscience and psychiatry encounter challenges related to the limited availability of high-quality annotated datasets and training-induced biases, both of which compromise generalizability [22]. This underscores that effective diagnostic use of AI requires not only architectural advancement but also the development of robust methodological foundations—from the creation of open, validated datasets to the formulation of independent testing protocols. It can be inferred that integrating multiple data modalities—audio, text, and visual signals—significantly enhances the precision and reliability of early psychiatric diagnosis. However, accurate identification is merely the starting point. Early detection does not translate into improved outcomes unless timely support is provided. This is where AI-powered technologies designed for therapeutic support and ongoing monitoring come into focus.

#### 3.2.2. Therapeutic Support and Monitoring

A growing number of AI-driven interventions are designed to offer patients real-time support. These technologies aim to reduce barriers stemming from workforce shortages and the geographic inaccessibility of mental health services. Among the most prominent examples are intelligent chatbots grounded in the principles of cognitive behavioral therapy (CBT), which are capable of delivering psychological assistance in real-time contexts. The use of such systems has been associated with statistically significant reductions in symptoms of anxiety and depression. A review of recent pilot and randomized studies indicated that AI-powered bots—such as Woebot and Youper—can contribute to short-term symptom reduction of up to 43% for anxiety and 48% for depression, with user engagement rates reaching 76% and therapeutic alliance scores averaging 3.03 on the WAI-SR scale [14], suggesting a high level of patient receptivity.

However, these findings are derived from studies with considerable variability in design and duration, often lacking control groups and blinding procedures. While the self-report instruments used (PHQ-9, GAD-7, and WAI-SR) are validated, they remain susceptible to subjective interpretation and short-term fluctuation. Although the effects achieved statistical significance, they do not always meet thresholds for clinical relevance and warrant direct comparison with traditional CBT. The potential of multimodal interfaces (text, voice, and video) is noted in several publications, yet systematic verification of their added value is still lacking.

The relevance of chatbot interventions as a form of autonomous patient support is substantiated by practical evidence. In a two-week, unblinded randomized controlled trial involving 70 university students, using Woebot at least five times per week led to a mean reduction of 2.58 points on the PHQ-9 scale (*p* = 0.017; Cohen’s d = 0.44) [23]. Reported user satisfaction reached 4.3 out of 5, indicating a high level of subjective engagement. Nonetheless, the study was limited by its brief duration, the absence of objective behavioral metrics, and the use of a passive control condition (e-book), all of which constrain the generalizability of the findings. The observed effect size, while statistically significant, remained moderate in terms of clinical relevance and warrants replication in more representative cohorts. Direct comparison with traditional CBT is further complicated by methodological and temporal discrepancies between interventions.

A complementary example is offered by a retrospective analysis of data from 2061 users of the Wysa AI application during the COVID-19 pandemic. The study revealed statistically significant reductions in depressive and anxiety symptoms, with correlation coefficients of r = 0.569 (PHQ-9) and r = 0.562 (GAD-7), both at *p* < 0.001 [24]. On average, users completed 29 digital sessions over the first 15 days, with the highest engagement observed in modules targeting anxiety, sleep, and self-compassion. Despite promising outcomes, the absence of a control group, the anonymized nature of the data, and the high variability in user engagement complicate efforts to evaluate the sustainability and clinical robustness of the effects. These findings appear to reflect short-term symptom shifts under pandemic-induced psychological strain and necessitate confirmation through more rigorously controlled and systematically designed studies.

The ability to detect crisis situations before users explicitly report them has become a defining frontier in the evolution of AI-powered mental health systems. According to an internal report by Wysa, based on engagement data from 19,000 users across 99 countries, approximately 5.2% of users exhibited crisis indicators, and in 82% of those cases, the system identified the potential threat prior to user confirmation [25]. Despite this, only 2.4% of individuals reached out to professional help lines, while 49.2% followed the platform’s recommended safety plan, and 46.6% engaged with breathing and stabilization exercises. While these figures suggest the prospective utility of automated algorithms for crisis response, the findings are derived from an unpublished corporate report lacking peer review or methodological standardization. As such, they should be interpreted with caution and not construed as clinical evidence of efficacy.

An alternative and conceptually advanced approach is exemplified by the FedTherapist project, which utilizes federated learning to enable continuous monitoring of patients’ psychoemotional states. The system analyzes user-generated textual and speech inputs directly on personal devices, without transmitting sensitive data to a centralized server. In a pilot study involving 46 English-speaking participants (10-day observation via MTurk and LOUO cross-validation), the model achieved a 0.15 improvement in the AUROC and an 8.21% reduction in the MAE for predicting depression, anxiety, stress, and mood levels, outperforming baseline methods [26]. Although the model demonstrated high accuracy under experimental conditions and adhered to ethical protocols, the results must be interpreted in light of important limitations: a small and non-representative sample, a brief observation period, and the absence of validation in real-world clinical environments. As of publication, the system remains at the prototype stage and has not been integrated into electronic health records (EHRs) or telemedicine platforms.

Summary characteristics of the included empirical studies are presented in Table 2.

Despite high user engagement and encouraging short-term outcomes, the broader scalability of such systems remains hindered by the lack of longitudinal trials, heterogeneity in evaluation methodologies, and unresolved concerns regarding data privacy and legal integration within healthcare infrastructures. To date, randomized long-term trials remain scarce, and the applied efficacy metrics are often inconsistent across studies. As a result, most AI-enabled interventions remain outside the bounds of formal medical systems capable of assessing their durability in real-world clinical environments.

Nonetheless, these technologies reveal the prognostic potential of AI—extending beyond therapeutic support toward the early anticipation of symptom exacerbation and individualized risk trajectories, well before the manifestation of overt clinical pathology.

#### 3.2.3. Predictive Models

Some patients seek help only after symptoms have already manifested. But what if we could anticipate clinical deterioration before it occurs? This is precisely where predictive AI models come into play. These technologies integrate heterogeneous data sources—ranging from structured medical records and clinical documentation to behavioral indicators passively captured via mobile devices—thereby enhancing the precision of individualized risk assessment for outcomes such as suicidal behavior, relapse, and treatment non-adherence. On average, these models have demonstrated predictive accuracies ranging from 70% to 85%, with AUC-ROC scores approaching 0.90, indicating strong discriminatory capacity in stratifying patients by risk of depression and other psychiatric disorders [27].

One of the most large-scale and compelling examples of artificial intelligence applied to suicide risk prediction is the Army STARRS project, which analyzed data from 53,769 psychiatric hospitalizations of U.S. military personnel between 2004 and 2009. The model, based on elastic net regression and trained on 421 features—including sociodemographic factors, psychiatric history, medication use, and criminal records—demonstrated strong stratification performance: 52.9% of all suicides occurred within the top 5% of individuals identified as highest risk (AUC = 0.85). Elevated risk was particularly pronounced during the first 30 days post-discharge, especially among those with combat exposure (HR = 2.4; 95% CI: 1.3–4.5) and active psychiatric conditions (HR = 3.1; 95% CI: 1.5–6.4) [28]. However, it is important to note that the model lacked interpretability mechanisms—no explainable AI (XAI) tools such as SHAP or LIME were applied, and key predictors were not contextualized in clinical terms. Validation was conducted internally (10-fold cross-validation), with no external dataset used, thereby limiting generalizability. Moreover, there is no evidence of the model’s deployment in real-world clinical workflows; it remains unclear whether predictions influenced surveillance strategies or decision-making processes. Nevertheless, the study illustrates the potential of machine learning to identify small, high-risk subpopulations—an asset that could support targeted intervention strategies.

Another promising example of predictive modeling was presented in a Chinese study involving patients with bipolar disorder (*n* = 384). A nomogram was constructed based on logistic regression, incorporating key clinical and demographic predictors: the Social Dysfunction Screening Scale (SDSS), sleep quality (PSQI), history of suicidal behavior, frequency of outpatient visits, and receipt of electroconvulsive therapy (ECT). Internal validation on the training set (*n* = 303) yielded high predictive accuracy (AUC = 0.924), whereas performance on an independent external test set (*n* = 81) declined to a moderate level (AUC = 0.741) [29]. The model offers a visual interpretive interface through the nomogram, which enhances its clinical usability. However, the application of modern explainable AI (XAI) techniques—such as SHAP or LIME—was not reported, and the contribution of individual variables to personalized predictions remains insufficiently explained. No information was provided regarding clinical implementation or how the predictions are used by practitioners in real-world settings. Moreover, the model was not evaluated over extended time intervals (e.g., 18–24 months), which raises concerns about its long-term robustness. Despite these limitations, the study demonstrates a high degree of interpretability and technical accessibility, particularly due to its reliance on simple, clinician-friendly variables. This makes the model potentially attractive for routine use, although its scalability and technological maturity remain limited.

Further evidence of predictive potential was demonstrated in a South Korean study (*n* = 330) that combined structured data from electronic health records (EHRs) with unstructured clinical notes—including psychological assessments, intake reports, and nursing documentation—processed using latent Dirichlet allocation (LDA) for topic modeling. This multimodal integration significantly improved predictive accuracy: the AUROC increased from 0.784 to 0.946 when all data types were included. However, external validation on an independent cohort (*n* = 4391) revealed a marked decline in performance, with the AUROC dropping to 0.616, highlighting the model’s limited transferability and its dependency on localized data structures [30].

Algorithmic transparency remained low, as no explainable AI (XAI) tools were implemented and the interpretability of contributing factors was restricted. Moreover, no information was provided regarding clinical integration or longitudinal performance assessment. As such, the model is currently classified as a research-stage prototype requiring further refinement before scaling or real-world application.

A summary of the key characteristics, architectures, and limitations of predictive models is presented in Table 3.

In summary, one of the most prevalent limitations of predictive models lies in their context-dependent accuracy: performance tends to deteriorate markedly during external validation, with even well-trained models losing predictive power beyond their original training cohorts. Second, such models are often highly sensitive to local data formats, resulting in reduced reproducibility when applied across institutions or countries. Third, interpretability remains limited—most algorithms lack integrated explainable AI (XAI) mechanisms, and clinicians are seldom provided with transparent justifications for decision-making. Moreover, the clinical impact of these models is rarely assessed; their influence on patient management strategies remains largely undefined. Collectively, these issues point to a low level of technological maturity, with most solutions still confined to the prototyping stage.

What is needed is a shift from static, one-time predictions toward dynamic, context-aware patient support. This appears to be an emerging direction for some telepsychiatry initiatives, which are exploring the integration of AI for continuous monitoring and adaptive care delivery, although current evidence remains limited.

### 3.3. Perspectives of Telepsychiatry

Modern telepsychiatry has begun to show promise in addressing shortages in the psychiatric workforce, geographic barriers, and inequities in access to mental healthcare, though robust empirical validation is still needed to confirm these benefits at scale. It is essential, however, to distinguish between the value of remote communication itself—such as video conferencing, scheduling flexibility, and reduced access barriers—and the added value of artificial intelligence as a functionality-enhancing layer. The integration of AI into digital platforms creates opportunities to automate key processes, from screening and triage to monitoring and decision support, thereby enabling more adaptive and personalized interactions. This is particularly relevant in regions with critically low psychiatrist-to-population ratios, where there is fewer than one specialist per 100,000 people. In such contexts, the increasing adoption of digital modalities can largely be attributed to this underlying structural deficit. For instance, in India, a systematic review has outlined several promising trajectories for AI-enhanced telepsychiatry—including speech and behavior analysis, NLP modules, and recommender systems—with a strong emphasis on the need for local adaptation and model validation [13]. Notably, most of these approaches remain at the stage of conceptual development or prototyping.

At the same time, the COVID-19 pandemic served as a powerful catalyst for the expansion of telepsychiatric channels. At VCU Health in the United States, remote consultations rapidly became the predominant mode of service delivery: in April 2020, telepsychiatric visits accounted for 92% of all encounters and subsequently stabilized at approximately 80%. Patients reported high levels of satisfaction with the ability to receive care in a comfortable and safe home environment, while clinicians emphasized the more “humanizing” quality of video visits compared to in-person interactions conducted under strict infection control protocols. Notably, even among elderly patients, no reduction in access to telemedicine services was observed [31].

**Figure 2:**
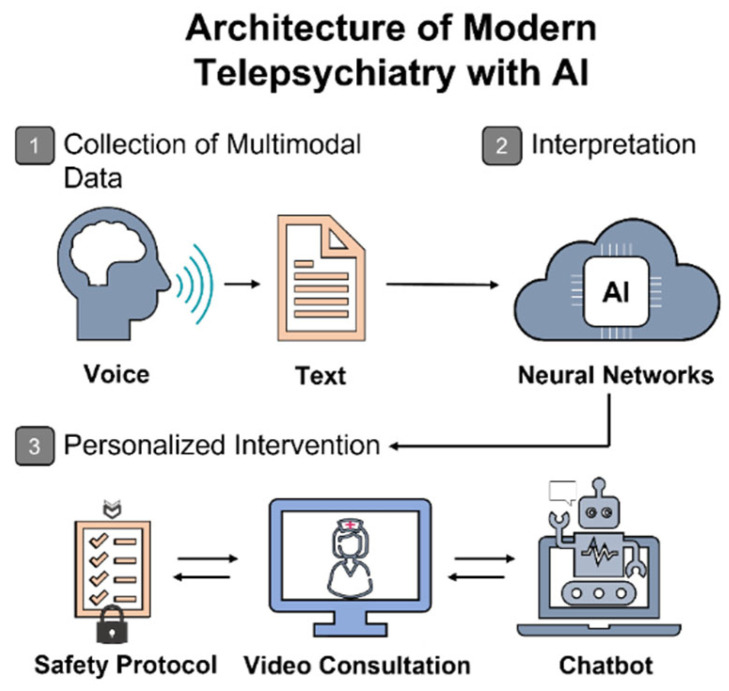
Conceptual framework of AI-enhanced telepsychiatry services. The illustration outlines the operational flow of AI-integrated telepsychiatry, beginning with the acquisition of patient-generated multimodal data (e.g., speech and text), followed by algorithmic interpretation via neural networks, and culminating in a personalized clinical response. This includes real-time interventions such as video consultations, chatbot-guided interaction, and adaptive safety protocols tailored to individual needs.

The emerging paradigm of personalized telepsychiatry is increasingly structured around a three-tiered architecture: (1) acquisition of multimodal data—speech, text, behavioral patterns, physical activity, smartphone usage, and physiological signals; (2) algorithmic interpretation through ensemble learning, feature transformation, and selection of key predictive variables; (3) generation of therapeutic recommendations and dynamic, adaptive monitoring (see Figure 2).

It is essential to delineate the respective contributions of each component: while telemedicine infrastructure provides the remote communication channel and facilitates continuous data collection, it is the AI layer that performs signal interpretation and prediction of clinical-state transitions. Studies have shown that models integrating data from wearable devices and smartphones can effectively estimate the severity of depressive symptoms. By combining sensor-based metrics—such as electrodermal activity (EDA), heart rate variability (HRV), temperature, motion, and sleep—with smartphone-derived indicators like screen activity, geolocation, call logs, and app usage, the researchers developed a model capable of predicting HDRS-17 depression severity with a correlation of r = 0.7 and a mean absolute error (MAE) of 3.88 (under time-split validation). The most informative predictors included activity level, phone interaction frequency, skin conductance, and HRV. The system demonstrated high user adherence: after resolving initial technical issues, over 90% of participants wore the sensors on a daily basis [32]. These findings suggest that telepsychiatry has the potential to move beyond traditional video consultations toward dynamic, adaptive digital accompaniment—an especially valuable asset for managing chronic affective disorders and high relapse risk. Moreover, such architectures may enhance patient autonomy by reducing the need for external prompts and increasing engagement in care. Nonetheless, as noted by the authors, the study was conducted at a pilot stage: the sample size was small (*n* = 31), symptom variability was limited, and while predictive accuracy was acceptable, it did not reach a clinically reliable threshold.

Although telepsychiatry expanded rapidly during the COVID-19 pandemic, perceptions of its effectiveness and acceptability remain ambivalent. A multinational study by Sheriff et al. (2023) [33], encompassing 1798 participants from the United Kingdom and Italy, revealed substantial discrepancies in attitudes across stakeholder groups—namely, patients, caregivers, and clinicians. While nearly 60% of users found remote consultations convenient for routine care, only 12.4% expressed willingness to transition fully to online formats. Moreover, in 58.5% of cases, the choice of consultation modality was determined by clinicians without incorporating patient preferences. Fewer than 30% of physicians considered telepsychiatry acceptable for initial diagnostic assessments, particularly in acute cases such as psychosis, suicidality, or severe agitation. The study highlighted several persistent barriers: limited patient involvement in decision-making, concerns about confidentiality, and poor suitability of remote care for acute psychiatric presentations. It is important to note that the study was conducted at an advanced observational stage but included only four clinical sites (two in each country), with evident underrepresentation of ethnic minorities and male participants. Additionally, systematic selection bias may have occurred, as individuals with low digital literacy could have been excluded from the sample. These factors limit the generalizability of findings but do not diminish their value: such evidence is critical for the design of equitable, adaptive, and consensus-driven telepsychiatry strategies.

In sum, the convergence of telepsychiatry and artificial intelligence holds significant promise for overcoming infrastructural and workforce constraints while fostering a more flexible, context-sensitive, and patient-centered model of care. Yet the realization of this potential depends on a set of interlocking prerequisites: institutional recognition, clinical validation, regulatory integration, and resilient digital infrastructure. Only under these conditions can AI have the potential to evolve from an experimental adjunct into a clinically integrated component of psychiatric care—one that meaningfully contributes to the individualization of care and the expansion of mental health service accessibility.

## 4. Discussion

### 4.1. Ethical and Legal Risks

At the intersection of psychiatry and artificial intelligence, what emerges is not simply a technological breakthrough but an ethically and legally intricate landscape defined by profound dilemmas. This multidimensional complexity has been repeatedly underscored in recent systematic reviews [34], which highlight autonomy, transparency, justice, and trust as particularly acute challenges in mental health contexts. Unlike other medical fields, psychiatry places questions of personal identity and social vulnerability at the very center, such that even minor algorithmic deviations may lead to disproportionate consequences—from unwarranted patient labeling to the exacerbation of existing social inequities [35]. Our findings both corroborate and extend these previous observations, offering empirical evidence of such risks across a wide range of clinical scenarios and AI model architectures.

#### 4.1.1. Violation of Autonomy and Justice

Even when highly accurate, algorithms may systematically reproduce inequalities embedded in the source data—and psychiatry is particularly susceptible to such effects. For example, at the University Medical Center Utrecht, gender bias was identified in an AI model predicting repeat benzodiazepine prescriptions: the Disparate Impact value was 0.793, indicating a consistent advantage for male patients. The implementation of a reweighing strategy significantly reduced this bias without a notable loss in accuracy (balanced accuracy: 0.834 → 0.830; F1-score: 0.843 → 0.839). This study, which demonstrated the feasibility of mitigating algorithmic bias using real-world psychiatric data, sends a clear message: fairness does not arise “by default”—it must be deliberately designed [36]. Algorithmic discrimination may manifest not only along gender lines but also in relation to age, ethnicity, educational background, or even the language used to interact with the system—and often remains undetected in the absence of dedicated fairness audits. This is why addressing bias requires not only technical instruments (e.g., reweighing, adversarial debiasing, and Fairlearn) but also mandatory procedures for evaluating fairness in the context of social vulnerability. Yet even this is insufficient: without formalized legal accountability—who bears responsibility for an error, the clinician or the developer? —any adjustments to the code will remain merely technical fixes. Without a clearly defined agent of responsibility, no genuine ethics is possible.

The broader societal context is equally critical. According to a representative survey of 2060 respondents from the United States and the United Kingdom, conducted as a randomized comparison between AI- and DSM-based diagnostics, the most frequently cited concerns included risks of discrimination, psychological distress following diagnosis, disruption of self-perception, and difficulty in communicating the diagnosis to others—all scoring above 4.5 on a 7-point scale. Paradoxically, DSM-based diagnosis elicited greater anxiety than the hypothetical AI algorithm, particularly in terms of communicability, emotional strain, and stigmatization (*p* < 0.05) [37]. Yet this is not an endorsement of AI—it reflects a deeper fatigue with models that exclude the patient from the process. An algorithm, even if explainable, does not resolve the fundamental question: who makes the decision, and who is held accountable? As long as AI systems remain faceless, they cannot inspire trust or offer protection. Genuine justice requires not only technical correction but a normative framework—one in which every diagnosis comes not only with an explanation but with a guarantee of responsibility.

#### 4.1.2. Privacy

Psychiatric data are inherently vulnerable—not merely reflecting symptoms but encompassing the most intimate dimensions of personhood: voice, text, behavioral patterns, lexical choices, and emotional states. In mobile environments, such data are under constant threat. A review of 27 widely used mental health apps revealed that 96% leaked personal information, 15 stored sensitive data in unencrypted form, and 20 posed critical security risks [38]. Particularly alarming are the risks of re-identification and deanonymization: through metadata and user IDs, individual profiles can be reconstructed even under the appearance of anonymity. While technical solutions such as differential privacy and federated learning have been proposed, they are rarely implemented in practice. Developers continue to rely on insecure channels and weak encryption protocols, and privacy policies often require graduate-level literacy to comprehend. Who bears responsibility for such vulnerability remains an unresolved question. In the absence of Privacy Impact Assessments (PIAs) and enforceable legal frameworks, patients are left defenseless against systems that process the most private facets of their identity.

#### 4.1.3. Opacity of Algorithmic Decision-Making

One of the most persistent challenges in applying AI to psychiatry is the opacity of algorithmic decision-making. Unlike a clinician, who can justify a diagnosis through transparent reasoning, most models function as “black boxes”, offering no insight into which features contributed to a given classification or prediction. This issue is particularly acute in psychiatry, where the input data often comprise behavioral, linguistic, and affective markers—domains highly susceptible to interpretive ambiguity. A systematic review of ethical risks [39] underscores that opacity not only limits verifiability but also undermines clinical interaction, erecting a barrier to meaningful informed consent. Even the use of explainable AI techniques—such as SHAP, LIME, or attention-based mechanisms—does not ensure interpretability at a level comprehensible to patients. In the absence of regulatory requirements for explainability and algorithmic auditing, such decisions remain effectively beyond the control of both clinicians and patients.

This lack of transparency is directly linked to the risk of harm: opaque models hinder error detection and increase the likelihood of overdiagnosis, misclassification, or inappropriate treatment allocation. As highlighted in a recent narrative review [40], ethical considerations in the application of AI to mental health underscore the imperative for transparency and explainability, as the absence of interpretability not only introduces new risks but also undermines patient trust and autonomy. In the context of stigmatized psychiatric diagnoses, the consequences may be not only psychological but also social—ranging from erosion of trust to the restriction of individual rights. Of particular concern is the absence of compensation mechanisms in cases of harm: neither patients nor clinicians are afforded assurances that an erroneous decision can be contested, reversed, or documented within a legal framework.

In this context, opacity emerges not merely as a technical limitation but as a normative failure—undermining trust, constraining autonomy, and reinforcing epistemic inequality between actors within the clinical encounter.

#### 4.1.4. International Ethical Frameworks and Their Implementation Gap

Several international organizations, including the WHO and UNESCO, have issued normative frameworks for the application of AI in medicine, aimed at mitigating core ethical risks—namely, autonomy, beneficence, non-maleficence, justice, transparency, and accountability [41,42]. These documents place particular emphasis on the protection of vulnerable populations, including individuals with mental health disorders, and highlight the need for routine algorithmic audits, multidisciplinary oversight, and meaningful informed consent. In practice, however, these principles rarely translate into enforceable mechanisms. In many countries, relevant regulations remain under development or exist only in the form of non-binding guidelines—for example, the EU Ethics Guidelines for Trustworthy AI [43] and the U.S. Blueprint for an AI Bill of Rights [44]. This underscores the fact that in the absence of mandatory national strategies and legal standards for explainability, even well-trained models may become sources of uncertainty—and, at times, of risk.

**Figure 3:**
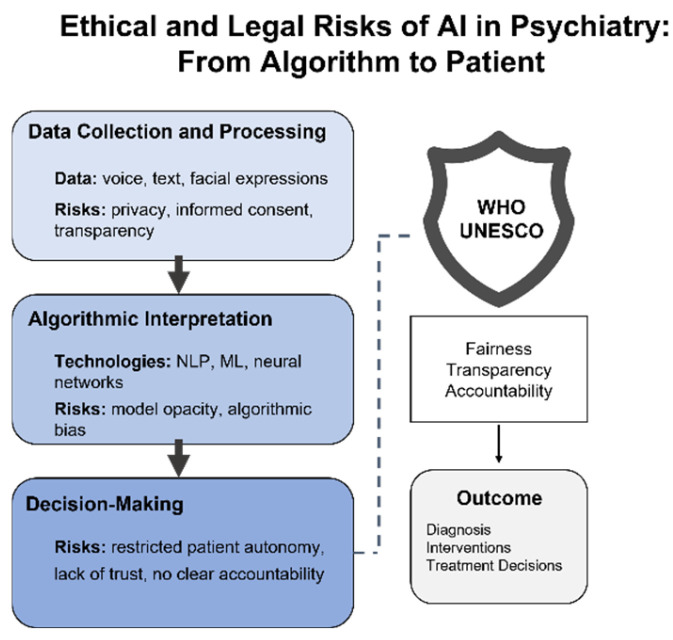
Ethical and legal risks of AI in psychiatry: from algorithm to patient. This conceptual diagram outlines the ethical risks at each stage of AI implementation in psychiatry—from data collection and algorithmic interpretation to decision-making. It also highlights the guiding role of international frameworks such as those developed by WHO and UNESCO, emphasizing fairness, transparency, and accountability in clinical outcomes.

Thus, the ethical and legal risks of AI in psychiatry are not abstract constructs but tangible realities that implicate patient identity, equity, and safety. Without built-in accountability, transparency, and protection mechanisms, any AI system risks not only forfeiting public trust but also exacerbating preexisting forms of vulnerability. The question is no longer whether algorithms can be trusted but whether the healthcare system can guarantee that trust. Figure 3 illustrates ethical risks across all stages of AI implementation—from data collection and model architecture to decision-making and clinical outcomes—underscoring the critical role of institutional safeguards.

### 4.2. Technological and Clinical Limitations

While ethical risks delineate the external boundaries of acceptability, technological and clinical limitations establish the internal constraints on the real-world applicability of AI systems. It is precisely at this intersection that the disparity between theoretical potential and operational resilience becomes most pronounced. During internal validation, neural network models—such as BERT or ResNet—may achieve remarkably high performance metrics, with AUC values reaching up to 0.98. However, when subjected to external validation, their accuracy frequently declines by as much as 6–22.8% [45]. This discrepancy stems not only from limited sample sizes but also from internal fragmentation within the datasets themselves. Specific examples of this external-performance degradation are documented in Supplementary Table S3.

Even in large-scale studies such as PREVENT (N = 6.6 million patients across 46 cohorts), critical variables were missing for substantial subgroups: race or ethnicity data were absent in 4–5% of cases; HbA1c values were unavailable for 70–75% of non-diabetic patients; and the Social Deprivation Index (SDI) was recorded for fewer than one-third [46]. This demonstrates that even with impressive sample sizes, representativeness may remain limited—particularly for age-, ethnicity-, and condition-specific subpopulations—thereby undermining the generalizability of models when applied to clinical settings. Older adults, ethnic minorities, and individuals with chronic or complex conditions are frequently overlooked by these systems, creating what has been termed an “invisible data crisis”. Algorithms trained on such foundations not only lose precision but risk reinforcing systemic distortions—especially in relation to marginalized or multifactorial patients.

Beyond algorithmic and data-related limitations, the issue of clinician trust remains unresolved. According to a survey conducted by the Alan Turing Institute in the United Kingdom, 29% of physicians reported using AI tools in their practice over the past 12 months, and more than half (52%) expressed optimism regarding its role in healthcare. Nonetheless, nearly one-third (32%) admitted that they do not fully understand the risks associated with AI implementation [47]. The primary sources of skepticism include insufficient algorithmic transparency, the inability to communicate decision logic to patients, and a lack of validated clinical evidence. Equally salient are interface-related barriers: cluttered visualizations, absence of standardized result formats, and unintuitive user experiences may hinder interpretation and reduce clinicians’ willingness to rely on the algorithm—even when its accuracy is demonstrably high.

**Figure 4:**
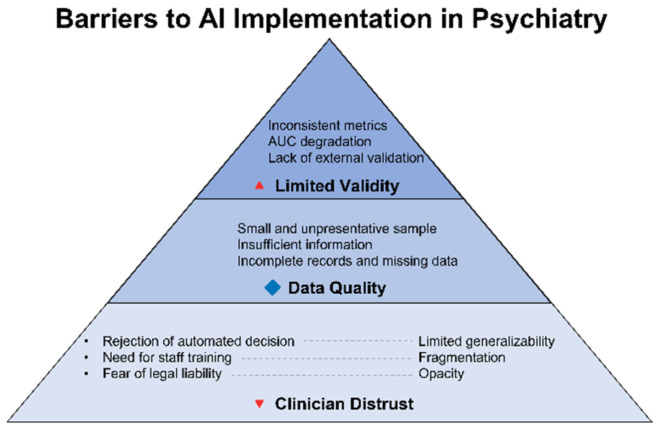
Technological and clinical limitations of AI in psychiatry: from data gaps to clinical uncertainty. This diagram illustrates the key limitations that hinder the clinical applicability of AI in psychiatry. It highlights the cascade of challenges—from missing or fragmented training data and a lack of external validation to issues with algorithmic transparency and clinician trust. Together, these factors undermine the robustness and generalizability of AI-based models in real-world healthcare settings.

Figure 4 illustrates barriers to clinical implementation of AI in telepsychiatry—from clinician distrust to technical opacity and the lack of representativeness in training datasets. Despite advances in model architecture, failure to address these challenges will relegate AI to the status of an experimental tool, incapable of integration into routine clinical practice.

Ultimately, the issues outlined above do not negate the value of artificial intelligence—they underscore the need to fundamentally reconsider its methodological foundations. It is essential to recognize that technological robustness and clinical applicability cannot be reduced to algorithmic refinement alone. Without expanded and diversified training datasets, independent external validation, and algorithmic explainability, we risk developing systems that excel at a single task: performing well under conditions that do not exist in the real world. Even high performance metrics become insufficient if a model cannot deliver clinically meaningful interpretations in a form suitable for decision-making. In this context, explainability is no longer merely an ethical imperative—it becomes a technical prerequisite for integration into practice: without a clear explanation, there can be no clinical action.

### 4.3. Scientific Gaps, Methodological Deficiencies, and Future Research Directions

Despite the growing body of literature on the application of AI in psychiatry, our analysis reveals systemic shortcomings in both research design and methodological maturity. Aggregated metrics and model architectures have been reviewed in Section 3.1, Section 3.2 and Section 3.3 and summarized in Supplementary Table S3. While the field has undoubtedly progressed, a number of foundational limitations continue to impede clinical integration at the level of healthcare systems. These gaps demand not rhetorical declarations but targeted, methodologically rigorous work—particularly if the goal is clinically actionable AI rather than experimental prototyping.

Unlike previous reviews focused predominantly on technical performance benchmarks, our analysis draws attention to the persistent gap between model-level metrics and real-world clinical applicability—particularly with respect to interpretability, ethical soundness, and long-term sustainability. While we reaffirm earlier findings on user engagement with AI-powered chatbots [14], our study underscores the lack of rigorous evaluation frameworks and the absence of harmonized assessment standards. Furthermore, in contrast to broader telepsychiatry literature [15], our synthesis offers a more nuanced typology of model maturity and translational challenges, substantiated by empirical indicators.

First, there is a notable absence of multicenter randomized controlled trials (RCTs) capable of objectively confirming the effectiveness and safety of digital interventions under real-world conditions. Without controlled studies featuring extended follow-up periods, it is impossible to assess the durability of treatment effects, their impact on quality of life, the risk of relapse, or the potential adverse consequences of AI-based interventions. Most current publications rely on small sample sizes and internal validation, often lacking control groups or clinical blinding—rendering their findings ineligible for direct extrapolation. Overcoming the limitations of laboratory-level evidence will require a new wave of standardized multicenter RCTs featuring transparent study designs, harmonized metrics, and clinically meaningful endpoints.

Second, although explainability is widely acknowledged as a prerequisite for clinical applicability, operationalized standards for its evaluation remain poorly defined in the scientific literature. As a result, “explainability” often functions as a rhetorical claim rather than a verifiable property of the model. This not only impedes translation into clinical practice but also undermines the comparability of findings across studies. Bridging this gap requires the development of formalized criteria—from method selection to validated interpretability metrics—that can be incorporated into standardized protocols for assessing the effectiveness and reliability of AI systems in psychiatry.

Third, there is still a lack of systematic research on the transferability of AI models to vulnerable subgroups—including older adults, adolescents, and ethnic or linguistic minorities. The majority of publications rely on validation within homogeneous samples, without conducting stratified analyses. We do not yet know how these algorithms perform outside their source populations. Without accumulating evidence on cross-group stability in model performance, it is impossible to speak of scientific reliability or to plan for scalable implementation.

Fourth, the field of psychiatric AI still lacks a recognized scale for assessing the clinical maturity of models. This absence hinders cross-study comparisons, impedes systematic progress tracking, and makes it impossible to stratify technologies by their level of readiness. Without a formal equivalent to the Technology Readiness Level (TRL)—adapted to the specific demands of clinical medicine—the research community is left without a framework for distinguishing between experimental prototypes and models that have completed the full cycle of clinical validation.

Fifth, ethics remains largely absent from the design process of AI models—it is typically addressed post hoc, once the architectural framework has already been established. There is no institutionalized practice of “ethics by design”, which would entail embedding principles of fairness, transparency, and accountability at every stage—from data selection to the publication of performance metrics. This is not merely an ethical concern; it constitutes a gap in scientific methodology itself, which still lacks standardized criteria for evaluating the ethical integrity of a model. Isolated instances—such as the use of fairness toolkits or reweighing procedures [36]—remain the exception rather than the rule.

Finally, there is a complete lack of systematic research tracking the long-term effects of AI interventions in psychiatry. The durability of diagnostic and therapeutic outcomes, the evolution of patient trust, and the impact on the therapeutic alliance all remain beyond the scope of empirical verification. Most RCTs are limited to observation periods of just 2–4 weeks [23], whereas psychiatric care demands time horizons measured in months and years. Without assessing delayed effects, risks of trust attrition, or relapse dynamics, it is impossible to evaluate the clinical viability of AI-based solutions under real-world conditions. This is not merely a research gap—it is a foundational deficit that renders the construction of a sustainable evidence base impossible.

These unresolved issues delineate key directions that demand priority in future research and the establishment of a robust scientific foundation for subsequent clinical integration.

### 4.4. The Potential of AI and Its Actual Clinical Applicability

Contemporary experience with the implementation of artificial intelligence in psychiatry demonstrates that technological advances, in and of themselves, do not guarantee clinical integration. Even the most advanced solutions remain at the stage of prototypes unless they are embedded within the institutional infrastructure, supported at the organizational level, and accompanied by changes in both administrative and educational practices. Illustrative examples from a pilot project—such as automated triage systems and digital assistants for therapeutic support—clearly indicate that effectiveness is determined by the flexibility of clinical workflows, the readiness of teams for interdisciplinary collaboration, the maturity of IT environments, the transparency of algorithms, and the incorporation of ethics-by-design principles from the outset of development. Ultimately, it is the healthcare system’s capacity for comprehensive adaptation—ranging from technological innovation to organizational culture—that constitutes the decisive condition for transforming potential into practical outcomes. System-wide adaptation at all levels, from technological platforms to institutional ethos, is essential for this transition.

### 4.5. Practical Mechanisms for the Sustainable Integration of AI into Psychiatric Practice

Whereas previous sections have outlined the scientific, technological, and organizational gaps impeding the clinical maturity of AI in telepsychiatry, the present subsection offers a comprehensive framework for overcoming these challenges. The central focus is placed on institutional, educational, and ethical mechanisms that can transform innovation from a laboratory prototype into an integrated tool of psychiatric care.

The consistent integration of AI into healthcare does not begin with isolated pilot projects but with a deliberate restructuring of clinical workflows. It is insufficient to limit efforts to technical testing or one-off implementations; what is required is the embedding of AI into the very fabric of routine practice. At every stage—from initial piloting to large-scale deployment—a rigorous analysis of real-world use cases is conducted, with active engagement of clinical teams, ongoing feedback, and adaptive refinement of standard operating procedures. Only such an approach allows for the transition from “adding a tool” to the genuine transformation of clinical routines, where innovation ceases to be an external addition and instead becomes an intrinsic element of professional practice.

Ethical stewardship must transcend formal compliance. The establishment of dedicated AI ethics boards—multidisciplinary committees composed of clinicians, engineers, patient representatives, and legal experts—ensures not merely declarative but truly effective oversight. Their role is not limited to verifying compliance with principles of fairness, transparency, and accountability at the design stage but extends to continuous guidance throughout the entire product lifecycle. In this way, the “ethics-by-design” principle is realized as a living process: sustained dialogue, vigilant monitoring of changes, and proactive mitigation of professional and social risks long before large-scale implementation.

A reimagining of educational policy is equally strategic. Certification programs, mandatory training for all categories of healthcare professionals, and regular in-person and remote workshops, as well as targeted courses for IT specialists and administrative teams, must evolve from isolated initiatives into a seamless part of professional development. Crucially, these programs should be grounded not in abstract knowledge but in real-world scenarios, reflecting the dynamic nature of clinical tasks and the constantly evolving requirements of digital tools. Only then can the inertia of traditional training be overcome and a culture of conscious and safe AI use in clinical settings be cultivated.

The creation of living laboratories for collaborative action is of fundamental importance. Living-lab platforms are not merely assemblies of stakeholders but ongoing, functional working groups in which clinicians, developers, patients, and regulators co-create, test, and iteratively refine solutions in real time. This approach provides the flexibility to identify and address weaknesses prior to scaling and ensures that all participants share collective responsibility for outcomes. Here, shared decision-making, transparent communication, and deep stakeholder engagement are established as standard practice rather than exception.

Another systemic mechanism is the standardization and openness of data. The development of open, validated, and continuously updated datasets; the implementation of independent audit procedures; and the public dissemination of model quality and reproducibility assessments together constitute the bedrock of trust, both within the professional community and among patients. Open infrastructure not only facilitates the scaling of successful solutions but also allows for the prompt correction of deficiencies, thereby minimizing the risks of inefficiency or uncontrolled proliferation of suboptimal models.

It is vital to underscore that none of these mechanisms can function effectively in isolation. Sustainable integration is achieved only when all directions—ethical supervision, professional education, collaborative expertise, and transparent data verification—advance in parallel, reinforcing and amplifying one another. Such a synergistic approach ensures not merely the incremental evolution from pilot projects to mature products, but fosters the emergence of a new culture of digital psychiatry: open, accountable, and inherently self-improving.

In conclusion, the proposed framework is not a collection of disparate initiatives but a coherent system of coordinated efforts by institutions, professional and patient communities, regulatory bodies, and technology companies. Only such a holistic strategy can transform artificial intelligence from an object of research enthusiasm into a robust and ethically grounded instrument for sustainable psychiatric care.

## 5. Limitations

This review has several limitations that should be taken into account when interpreting the findings. Foremost among them is the high heterogeneity of the included studies: sample characteristics, clinical contexts, AI model architectures, data modalities, and validation methods vary substantially. This heterogeneity precludes the possibility of conducting a meta-analysis and limits the potential for the quantitative integration of the results.

The search strategy was limited to English-language sources, which may have led to the omission of relevant publications in other languages. Some of the included studies were available only in preprint format, posing potential risks associated with unverified information. In addition, the possibility of publication bias cannot be ruled out—namely, the underrepresentation of studies reporting neutral or negative results.

A formal risk-of-bias assessment was not performed, as the methodological heterogeneity of study designs precluded the use of a unified analytical tool. Potential limitations were instead addressed qualitatively and are further discussed in Section 4.2.

These factors underscore the need for future standardized empirical studies employing consistent reporting frameworks, independent validation, and stratified analysis of AI models in psychiatric research.

## 6. Use of Artificial Intelligence

During the preparation of this review, the language model ChatGPT (OpenAI, GPT-4o mini, desktop application v.1.2025.139.0) was used exclusively for editorial support. The model assisted with stylistic editing, refinement of phrasing, and verification of logical coherence across sections. No part of the analytical process—including screening, data extraction, or result interpretation—was performed automatically; all steps remained fully under the control of the researchers.

## 7. Conclusions

### 7.1. Summary and Outlook

Artificial intelligence in psychiatry remains a high-potential technology that, despite growing interest, has yet to be fully integrated into clinical practice. This review offers the first PRISMA-structured synthesis to concurrently address both empirical evidence and the normative–ethical landscape of AI implementation in telepsychiatry. Its objective is not only to consolidate fragmented literature but also to establish a thematic framework for evaluating the maturity of digital interventions and identifying structural impediments to their sustainable integration. Among the persistent deficits are the absence of multicenter clinical trials, a lack of longitudinal data, the underdevelopment of criteria for model interpretability, and limited representation of vulnerable populations.

The practical relevance of this review lies in its multi-stakeholder orientation. For clinicians, the findings offer a reference point for navigating available models and assessing their clinical applicability. For developers, it is demonstrated that further progress depends less on architectural innovation than on addressing challenges of linguistic adaptability, reproducible model transparency, and privacy-preserving learning frameworks. For regulators and decision-makers, the review outlines concrete priorities: developing certification levels for technological maturity, implementing fairness auditing mechanisms, and formalizing regulatory standards for both autonomous and hybrid AI systems. Telepsychiatry, in particular, calls for a systematic, normatively verifiable, and ethically grounded approach to AI integration—as a trustworthy component of complex digital health infrastructure.

The proposed research agenda is articulated around four interdependent priorities. First, the implementation of multicenter randomized controlled trials of at least one year in duration, with clinically meaningful endpoints. Second, the development of a TRL-Psy scale to classify models along a continuum from laboratory prototypes to real-world deployment, with mandatory validation of interpretability. Third, the creation of open-access multimodal corpora representing diverse regions and languages—crucial for assessing transferability and mitigating hidden bias. Fourth, the institutionalization of ethics-by-design principles and long-term monitoring of delayed effects of AI interventions among sensitive and high-risk populations.

### 7.2. Practical Recommendations for Clinical Practitioners and Policymakers

For the sustainable and meaningful integration of artificial intelligence into psychiatric care, it is advisable to adopt the following approach. Clinical teams should prioritize AI tools that have undergone independent external validation and demonstrate transparency in their decision-making logic. It is essential to integrate algorithm-generated recommendations into the structure of professional judgment, viewing them as a complementary—rather than definitive—source of information. This requires active participation in comprehensive educational programs, where training in the safe and effective use of digital solutions becomes not an isolated episode but an integral component of ongoing professional development.

From the perspective of regulators and policymakers, the introduction of mandatory procedures for external validation and independent auditing of all deployed AI systems is warranted, alongside the development of standards for transparency, explainability, and ethics by design to be implemented at both the design and maintenance stages of algorithmic solutions. Supporting initiatives for the creation and open dissemination of representative datasets is also critical for ensuring reproducibility and equity in diverse clinical and sociocultural contexts. Only the comprehensive implementation of these mechanisms can transform AI into a reliable, clinically mature, and ethically grounded instrument of contemporary psychiatric practice.

Only through coordinated progress across these domains can artificial intelligence evolve from demonstrative prototypes into a clinically robust, personalized, and equitable tool in psychiatric care.

## Figures and Tables

**Table 1:** The main approaches to the diagnosis and screening of mental disorders using AI.

Study	Approach	Data Type	Models	Validation		Accuracy (%)	F1 (%)	Sample Size/Classes	Maturity Level	Limitation
Chen Y. et al., 2024 [18]	NLP models for speech analysis	Audio	SVM, MLP, HuBERT, CNN, Transformer	Internal (k-fold CV); no external		94.1	Not reported	N = 270 2 classes	Moderate	Lab conditions only
Yin F. et al., 2023 [20]	Parallel architecture	Audio	Transformer + Parallel CNN	Internal (LOOCV); no external		Not reported	96.7	DAIC-WOZ = 4401 MODMA = 1321 2 classes each	Moderate	Potential leakage; no independent evaluation
Mahayossanunt Y. et al., 2023 [21]	Facial LSTM w/attention	Video	Window-Block LSTM + IG	Train/dev/test split + external test set	91.7	88.9	N = 474 2 classes	Prototype	Few severe cases; data closed	

**Table 2:** AI tools to support therapy and monitoring: architectures, metrics, and limitations of empirical research.

Study	Approach	Data Type	Models	Validation	Key Metrics	Sample Size/Classes	Maturity Level	Limitation
Fitzpatrick K. et al., 2017 [23]	CBT chatbot	Text	Decision tree + NLP	RCT (2 weeks, ITT), no external validation	PHQ-9 d = 0.44 (*p* = 0.017)	N = 70 2 arms	Trial stage	Short follow-up; small N; no blinding
Sinha C. et al., 2023 [24]	AI-chatbot + CBT	Text	ML + behavioral modules	Retrospective user analysis, no external validation	PHQ-9 r = 0.569; GAD-7 r = 0.562	N = 4541 continuous scores	Market product	No control group; anonymized data; generalizability unclear
Shin J. et al., 2023 [25]	FL-based CALL model	Text + speech + context	Fixed-BERT + MLP	LOUO (N = 46; 10 days), no external validation	AUROC = 0.746; MAE ↓ 8.21%	N = 46 users—regression outputs	Prototype	Short period; small sample; English only; no EHR integration

**Table 3:** Predictive AI models in psychiatry: architectures, data, validation, and limitations.

Study	Approach	Data Type	Models	Validation	Key Metrics	Sample Size/Classes	Maturity Level	Limitation
Kessler R.C. et al., 2015 [28]	Suicide risk post-discharge (Army STARRS)	EHR	Elastic Net + Survival Model	Internal (10-fold CV), no external validation	AUC = 0.85	N = 53,769 2 classes	Prototype	No XAI; no deployment
Zhang X. et al., 2025 [29]	Bipolar relapse prediction	Demographics, SDSS, PSQI, visits	Logistic + Nomogram	Internal (train: *n* = 301), external (test: *n* = 81)	AUC = 0.924 (train); 0.741 (valid)	N = 384 2 classes	Moderate	Small external set; short follow-up; no XAI
Lee D.Y. et al., 2022 [30]	Psychosis relapse prediction	EHR + clinical notes (NLP)	LASSO-LogReg + LDA	Internal (3-fold CV: *n* = 330), external (test: *n* = 4391)	AUROC 0.946 → 0.616 (ext.)	Int. *n* = 330; Ext. *n* = 4391 2 classes	Exploratory prototype	Heterogeneous DX; limited variable types; no PANSS; lacks XAI

## Data Availability

All the data used in this review are provided within the main text and Supplementary Materials (Tables S1–S4; Figure S1). No new primary data were generated in the course of this study.

## Supplementary Materials

The following supporting information can be downloaded at https://www.mdpi.com/article/10.3390/healthcare13111348/s1: Figure S1: PRISMA 2020 flow diagram illustrating the publication selection process. Table S1: Search strategy for each database. Table S2: Inclusion and exclusion criteria for publication selection. Table S3: Characteristics of empirical studies: model architecture, sample, metrics, and limitations. Table S4: PRISMA 2020 compliance checklist.

## Abbreviations

AI	Artificial Intelligence
AMA	American Medical Association
AUC	Area Under the Curve
AUROC	Area Under the Receiver Operating Characteristic Curve
CBT	Cognitive Behavioral Therapy
CI	Confidence Interval
EHRs	Electronic Health Records
GRU	Gated Recurrent Unit
HR	Hazard Ratio
JMIR	Journal of Medical Internet Research
LIME	Local Interpretable Model-Agnostic Explanations
MAE	Mean Absolute Error
NLP	Natural Language Processing
PREVENT	Predicting Cardiovascular Events Study
PRISMA	Preferred Reporting Items for Systematic Reviews and Meta-Analyses
RCT	Randomized Controlled Trial
SHAP	SHapley Additive exPlanations
SR	Short Revised (Working Alliance Inventory)
STARRS	Study to Assess Risk and Resilience in Servicemembers
SVM	Support Vector Machine
UNESCO	United Nations Educational, Scientific and Cultural Organization
WHO	World Health Organization
