# Supplementary material for: Telepsychiatry and Artificial Intelligence: A Structured Review of Emerging Approaches to Accessible Psychiatric Care
Source: Healthcare (Basel). 2025 Jun 5;13(11):1348. doi: 10.3390/healthcare13111348 (PMC12155282; doi:10.3390/healthcare13111348)
Supplement: Supplementary file 1 [file healthcare-13-01348-s001.zip › Figure S1. PRISMA 2020 flow diagram illustrating the publication selection process.pdf]

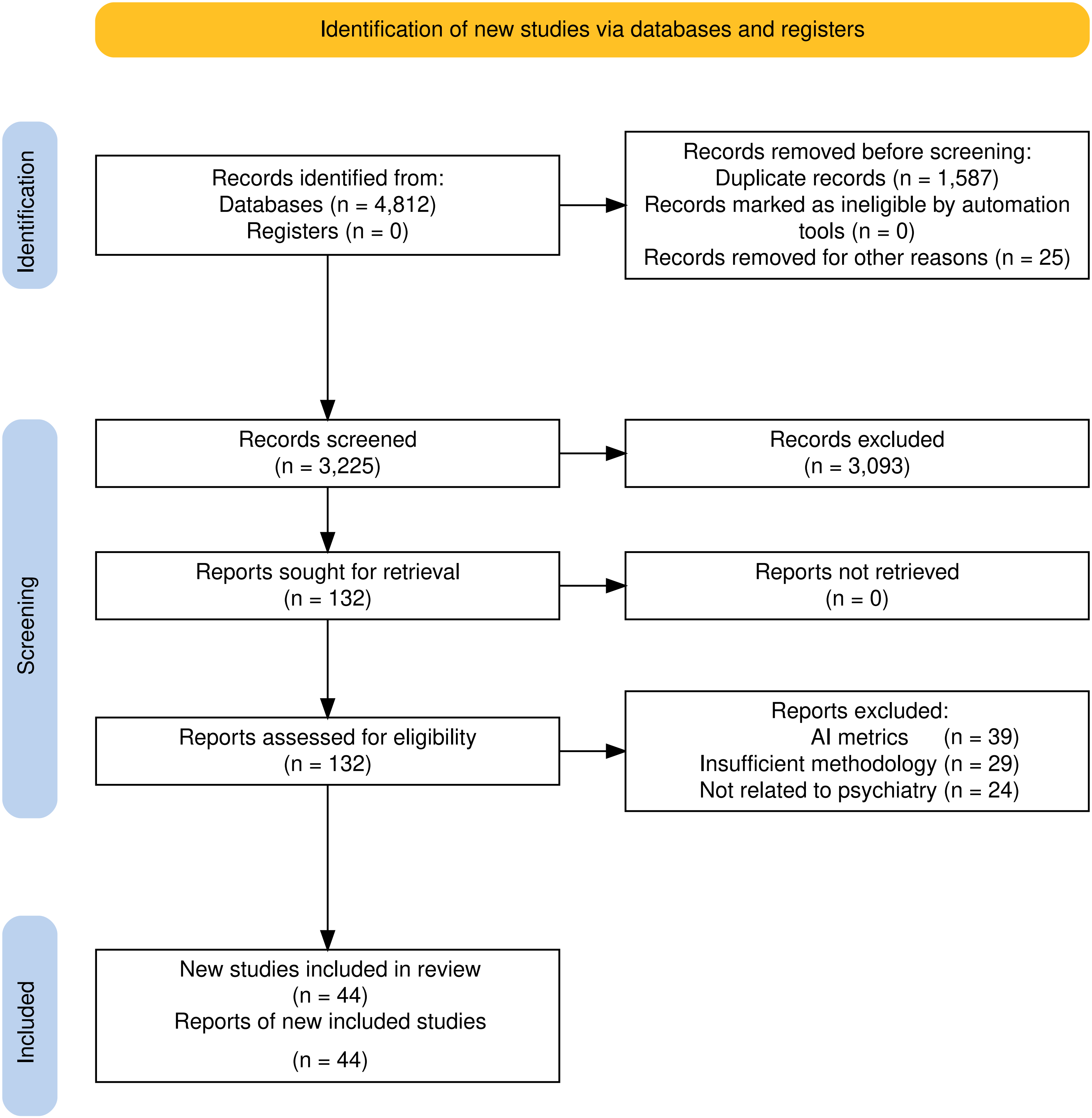
Identification of new studies via databases and registers
Identification
Records identified from:
Databases (n = 4,812)
Registers (n = 0)
Records removed before screening:
Duplicate records (n = 1,587)
Records marked as ineligible by automation tools (n = 0)
Records removed for other reasons (n = 25)
Screening
Records screened
(n = 3,225)
Records excluded
(n = 3,093)
Reports sought for retrieval
(n = 132)
Reports not retrieved
(n = 0)
Reports assessed for eligibility
(n = 132)
Reports excluded:
AI metrics (n = 39)
Insufficient methodology (n = 29)
Not related to psychiatry (n = 24)
Included
New studies included in review
(n = 44)
Reports of new included studies
(n = 44)
