# Supplementary material for: Telepsychiatry and Artificial Intelligence: A Structured Review of Emerging Approaches to Accessible Psychiatric Care
Source: Healthcare (Basel). 2025 Jun 5;13(11):1348. doi: 10.3390/healthcare13111348 (PMC12155282; doi:10.3390/healthcare13111348)
Supplement: Supplementary file 1 [file healthcare-13-01348-s001.zip › Table S1. Search strategy for each database.pdf]

Table S1. Search strategies and keywords

Table S1.1. PubMed

№	Search expression
**1**	("Artificial Intelligence"[Mesh] OR "Machine Learning"[Mesh]) AND ("Mental Health"[Mesh] OR "Psychiatry"[Mesh]) AND ("Telemedicine"[Mesh] OR "Digital Health")
**2**	("deep learning" OR "transformers" OR "neural networks") AND ("depression diagnosis" OR "anxiety monitoring")
**3**	((AI OR ML) AND (mental health OR psychiatry)) AND (chatbot OR CBT OR "cognitive therapy")
**4**	("natural language processing" OR NLP) AND ("suicidal ideation" OR suicide OR "self-harm")
**5**	("digital phenotyping" AND mood disorders AND smartphone)
**6**	depression AND machine learning
**7**	AI AND psychiatry
**8**	(machine learning AND suicide prediction)
**9**	("predictive modeling" AND "mental illness")
**10**	("mental health" AND (telepsychiatry OR telehealth) AND "artificial intelligence")

Table S1.2. Scopus

№	Search expression
**11**	TITLE-ABS-KEY(("artificial intelligence" OR "machine learning") AND ("psychiatry" OR "mental health") AND ("telemedicine" OR "telepsychiatry"))
**12**	TITLE-ABS-KEY(("natural language processing" OR NLP) AND depression AND diagnosis)
**13**	TITLE-ABS-KEY((AI OR ML) AND chatbot AND anxiety)
**14**	TITLE-ABS-KEY((wearable sensors AND depression) OR (mobile health AND AI))
**15**	TITLE-ABS-KEY((predictive models AND psychiatry) AND "suicide prevention")
**16**	TITLE-ABS-KEY(("digital psychiatry" AND "federated learning"))
**17**	TITLE-ABS-KEY(("deep learning" AND mood disorders AND smartphone))
**18**	TITLE-ABS-KEY((CBT chatbot AND mental health AND artificial intelligence))
**19**	TITLE-ABS-KEY(("explainable AI" AND mental health diagnosis))
**20**	TITLE-ABS-KEY((emotion recognition AND psychiatry))

Table S1.3. Web of Science

№	Search expression
**21**	TS=("artificial intelligence" OR "machine learning" OR "deep learning") AND TS=("psychiatry" OR "mental health") AND TS=("telemedicine" OR "telepsychiatry")
**22**	TS=(NLP OR "natural language processing") AND TS=("depression diagnosis")
**23**	TS=(CBT chatbot AND anxiety)
**24**	TS=(wearable sensors AND mood monitoring)
**25**	TS=("mobile mental health apps" AND "AI")
**26**	TS=("emotion recognition" AND psychiatry AND neural networks)
**27**	TS=(predictive modeling AND suicide risk)
**28**	TS=("telepsychiatry" AND "digital biomarkers")
**29**	TS=("deep learning" AND stress detection AND speech)
**30**	TS=("explainable AI" AND psychiatry)
**31**	TS=(EEG AND depression AND classification)

Table S1.4. PsycINFO

№	Search expression
**32**	("Artificial Intelligence" AND "Mental Disorders")
**33**	("Machine Learning" AND "Psychiatry")
**34**	("Digital Psychiatry" AND "Chatbot")
**35**	("Telemedicine" AND "AI-powered monitoring")
**36**	("Natural Language Processing" AND "Mental Health")
**37**	("Suicidal Ideation" AND "Predictive Modeling")

Table S1.5. arXiv\Frontiers

№	Search expression
**38**	AI psychiatry
**39**	"telepsychiatry AND NLP"
**40**	"mental health" AND "machine learning" AND depression
**41**	("deep learning" OR "transformer model") AND "suicide prediction"
**42**	"digital phenotyping" AND "bipolar disorder" AND smartphone
**43**	"emotion recognition" AND (EEG OR "facial expression") AND psychiatry
**44**	cat:cs.AI AND "depression detection"
**45**	cat:q-bio.NC AND ("cognitive state" OR "mental disorder") AND "AI"
**46**	abs:"federated learning" AND "mental health monitoring"
