# Supplementary material for: Telepsychiatry and Artificial Intelligence: A Structured Review of Emerging Approaches to Accessible Psychiatric Care
Source: Healthcare (Basel). 2025 Jun 5;13(11):1348. doi: 10.3390/healthcare13111348 (PMC12155282; doi:10.3390/healthcare13111348)
Supplement: Supplementary file 1 [file healthcare-13-01348-s001.zip › Table S2. Inclusion and exclusion criteria for publication selection.pdf]

Table S2. Inclusion and exclusion criteria.

№	Inclusion Criteria	Exclusion Criteria
**1**	Publications addressing the use of artificial intelligence (AI) in psychiatry, mental health, telemedicine, or digital psychiatry	Studies not directly related to AI in psychiatry (e.g., general reviews of digital technologies without a specific focus on mental disorders)
**2**	Original empirical studies employing methods such as machine learning, deep learning, natural language processing (NLP), multisensory analysis, or federated learning	Conceptual and methodological papers lacking an empirical foundation
**3**	Studies reporting quantitative performance metrics of AI models (e.g., accuracy, AUC, F1-score, recall) along with descriptions of model architectures	Publications lacking quantitative metrics, data analysis, or sufficient information about the applied models
**4**	Randomized controlled trials (RCTs), cohort studies, pilot trials, as well as retrospective and prospective observational studies with detailed information on sample and outcomes	Articles lacking details on study design, sample size, data characteristics, or validation procedures
**5**	Review articles (including systematic and scoping reviews) offering structured analysis, classification, identification of barriers, or methodological directions used in the analytical sections of this review	Non-peer-reviewed sources lacking methodological transparency or presented solely as short opinions or letters to the editor
**6**	Preprints (including arXiv) providing complete details on study design, applied models, sample size, and performance metrics	Preprints or non-indexed materials missing key information regarding study design or outcomes
**7**	Studies based on real-world AI applications in telepsychiatry and digital patient support (e.g., during the COVID-19 pandemic)	Papers describing hypothetical or conceptual scenarios without connection to clinical practice or real-world user data
