# Supplementary material for: Telepsychiatry and Artificial Intelligence: A Structured Review of Emerging Approaches to Accessible Psychiatric Care
Source: Healthcare (Basel). 2025 Jun 5;13(11):1348. doi: 10.3390/healthcare13111348 (PMC12155282; doi:10.3390/healthcare13111348)
Supplement: Supplementary file 1 [file healthcare-13-01348-s001.zip › Table S4. PRISMA 2020 compliance checklist.pdf]

Table S4. PRISMA 2020 Checklist: Assessment of Systematic Review Compliance.

№	PRISMA Item	Status	Author Comment
**1**	Title	Yes	Clearly reflects the aim and design of a systematic review.
**2**	Abstract	Yes	Structured; includes objectives, methods, results, and conclusions.
**3**	Introduction – Rationale	Yes	Global context and relevance are thoroughly explained.
**4**	Introduction – Objectives	Yes	Stated explicitly at the end of the introduction.
**5**	Protocol and Registration	No	Not registered — initially designed as a narrative review.
**6**	Information Sources	Yes	All databases and manual search methods are described.
**7**	Search Strategy	Yes	Search example provided; full strategies in Supplement.
**8**	Selection Process	Yes	Two-step screening by two reviewers; described in detail.
**9**	Data Collection Process	Yes	Manual extraction using standardized Excel tables (see S3).
**10**	Data Items	Yes	Architecture, sample size, validation, metrics — all explicitly listed.
**11**	Study Risk of Bias Assessment	No	Not conducted due to heterogeneity in design and reporting formats.
**12**	Effect Measures / Synthesis Methods	Yes	Narrative synthesis by theme; supported by figures and summary tables.
**13**	Results: Individual Studies	Yes	Performance metrics, model types, limitations — clearly presented.
**14**	Heterogeneity Assessment	Yes	Discussed in Limitations section (samples, methods, metrics).
**15**	Sensitivity Analyses	No	Not performed; meta-analysis was not applied.
**16**	Study Selection Flow	Yes	38 included papers, 13 empirical; process outlined and visualized (PRISMA).
**17**	Study Characteristics	Yes	Detailed in Supplementary Table S3: country, model, data, validation, etc.
**18**	Risk of Bias in Studies	Yes	Limitations analyzed narratively; no formal risk tool applied.
**19**	Synthesis of Results	Yes	Divided by task category; with tables and empirical examples.
**20**	Publication Bias	No	Not addressed; should be noted in Limitations section.
**21**	Certainty (GRADE)	No	Not assessed; acknowledged as limitation.
**22**	Interpretation	Yes	Scientific conclusions drawn per section.
**23**	Review Limitations	Yes	Language, preprints, heterogeneity, and lack of bias formalization acknowledged.
**24**	Conclusions	Yes	Clear synthesis of findings with barriers and gaps highlighted.
**25**	Funding	Yes	No funding received; disclosed.
**26**	AI Assistance	Yes	ChatGPT-4o used for editing and coherence, not for data extraction or interpretation.
